# The transcriptomic landscape of elderly acute myeloid leukemia identifies *B7H3* and *BANP* as a favorable signature in high-risk patients

**DOI:** 10.3389/fonc.2022.1054458

**Published:** 2022-11-24

**Authors:** Sara Villar, Beñat Ariceta, Xabier Agirre, Aura Daniela Urribarri, Rosa Ayala, David Martínez-Cuadrón, Juan Miguel Bergua, Susana Vives, Lorenzo Algarra, Mar Tormo, Pilar Martínez, Josefina Serrano, Catia Simoes, Pilar Herrera, Maria José Calasanz, Ana Alfonso-Piérola, Bruno Paiva, Joaquín Martínez-López, Jesús F. San Miguel, Felipe Prósper, Pau Montesinos

**Affiliations:** ^1^ Servicio de Hematología y Terapia Celular, Clínica Universidad de Navarra, Instituto de Investigación Sanitaria de Navarra (IDISNA), Pamplona, Spain; ^2^ CIBERONC Centro de Investigación Biomédica en Red de Cáncer, Pamplona, Spain; ^3^ Centro de Investigación Médica Aplicada (CIMA) LAB Diagnostics, Universidad de Navarra, Pamplona, Spain; ^4^ Program of Hematology-Oncology, CIMA, Universidad de Navarra, Pamplona, Spain; ^5^ Navarrabiomed, Fundación Miguel Servet, Pamplona, Spain; ^6^ Hospital Universitario 12 de octubre, Madrid, Spain; ^7^ Hospital Universitario y Politécnico la Fe, Valencia, Spain; ^8^ Hospital San Pedro de Alcántara, Cáceres, Spain; ^9^ ICO Badalona- Hospital Germans Trias i Pujol, Badalona, Spain; ^10^ Hospital General de Albacete, Albacete, Spain; ^11^ Hospital Clínico Universitario de Valencia, Valencia, Spain; ^12^ Hospital Universitario Reina Sofía, Córdoba, Instituto Maimónides de Investigación Biomédica de Córdoba (IMIBIC), Córdoba, Spain; ^13^ Hospital Universitario Ramón y Cajal, Madrid, Spain

**Keywords:** acute myeloid leukemia, elderly, transcriptomics, biomarkers, prognosis

## Abstract

Acute myeloid leukemia (AML) in the elderly remains a clinical challenge, with a five-year overall survival rate below 10%. The current ELN 2017 genetic risk classification considers cytogenetic and mutational characteristics to stratify fit AML patients into different prognostic groups. However, this classification is not validated for elderly patients treated with a non-intensive approach, and its performance may be suboptimal in this context. Indeed, the transcriptomic landscape of AML in the elderly has been less explored and it might help stratify this group of patients. In the current study, we analyzed the transcriptome of 224 AML patients > 65 years-old at diagnosis treated in the Spanish PETHEMA-FLUGAZA clinical trial in order to identify new prognostic biomarkers in this population. We identified a specific transcriptomic signature for high-risk patients with mutated *TP53* or complex karyotype, revealing that low expression of *B7H3* gene with high expression of *BANP* gene identifies a subset of high-risk AML patients surviving more than 12 months. This result was further validated in the BEAT AML cohort. This unique signature highlights the potential of transcriptomics to identify prognostic biomarkers in in elderly AML.

## Introduction

Acute myeloid leukemia (AML) remains a disease of the elderly, with the median age at diagnosis of 70 years old. While young and fit patients with AML may receive an intensive approach with chemotherapy and hematopoietic stem cell transplantation (HSCT) (1, 2) as consolidation, older or frail patients do not benefit from this strategy and receive less intensive and unfrequently curative approaches (3–5).

Current risk stratification in AML patients is based mainly on cytogenetics and the presence of common genetic aberrations (*NPM1*, *FLT3-ITD*, *CEBPA*, *RUNX1*, *ASXL1*, and *TP53* mutations) best exemplified in the ELN risk classification system (1). This classification has been validated for young and older but fit patients treated with intensive chemotherapy (1, 6) and defines 3 prognostic groups based exclusively on genetic data. In this context core binding factor (CBF) leukemias, *NPM1* and biallelic *CEPBA* mutations are considered of good prognosis, while complex or monosomal karyotype, *TP53*, *FLT3-ITD*, *RUNX1* and *ASXL1* mutations, other recurrent translocations and *KMT2A* rearrangements confer a poor prognosis. However, this classification is not validated for elderly patients treated with a non-intensive approach, and its performance seems to be suboptimal in this context (7).

In addition to molecular and clinical characteristics, alternative biomarker panels such as other somatic mutations and gene expression profiling have been proposed to refine risk classification in AML patients (8–12), providing models with a prognostic value. Clinical implementation of an improved AML risk classification model has the potential to aid in clinical decision-making including the indication of HSCT for patients with intermediate and adverse risk. However, the outcome of patients with specific cytogenetic and molecular abnormalities such as *TP53* mutations or complex karyotype is still disappointing, especially when both characteristics are present in the same patient, with virtually all patients relapsing soon after initial treatment (1, 13).

AML in the elderly remains a clinical challenge. On the one hand, comorbidities and general performance status are important factors limiting an intensive therapeutic approach, thus a careful multi-domain assessment should be ideally considered when deciding the best treatment option for an old patient with AML (14–16). On the other hand, the proportion of adverse genetic abnormalities such as high risk cytogenetics and *TP53* mutations is higher in the elderly (17, 18). Considering these clinical features, the prognosis of AML in elderly patients remains dismal, with a five-year overall survival rate below 10% (19, 20).

In the current study, we analyzed the transcriptome of 224 newly diagnosed elderly AML patients treated in the Spanish PETHEMA-FLUGAZA clinical trial, with the aim to define new prognostic groups in this population. The detailed results of treatment schedules, clinical outcomes with minimal residual disease (MRD) data, and genomic landscape of PETHEMA-FLUGAZA patients have been previously published (21–23).

## Methods

### Study design

The multicentric PETHEMA-FLUGAZA phase 3 clinical trial (NCT02319135) included a total of 283 elderly patients (> 65-year-old) diagnosed with *de novo* or secondary AML, who were randomized to receive FLUGA (n=141), consisting of 3 induction cycles with fludarabine and cytarabine followed by 6 consolidation cycles of reduced intensity FLUGA (riFLUGA), or AZA (n=142), 3 induction cycles with 5-azacitidine followed by 6 identical consolidation cycles (**Figure S1A** **).** Patients diagnosed with acute promyelocytic leukemia and ECOG ≥ 4 were excluded from the trial.

**Figure 1:**
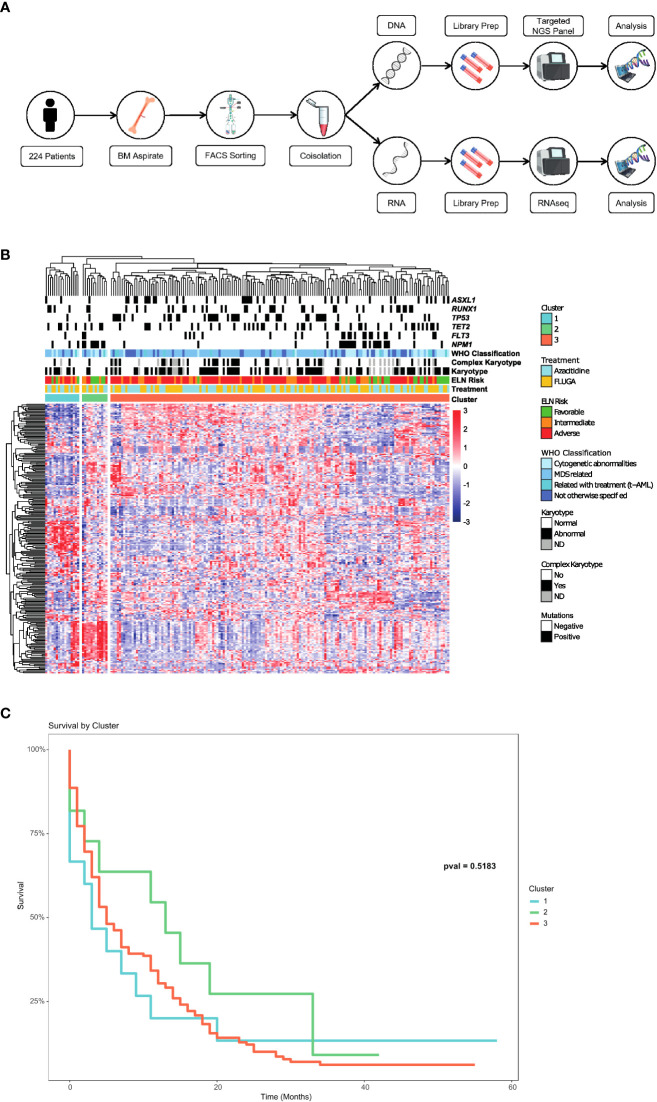
Identification of a long survival group beyond 12 months in elderly AML patients. **(A)** Scheme of the process carried out to obtain the RNAseq and mutation data through a myeloid NGS panel in AML samples of the PETHEMA-FLUGAZA clinical trial. **(B)** Unsupervised hierarchical clustering of the entire AML patient cohort using full transcriptional profiling, identifying 3 different groups. **(C)** Overall survival analysis of the 3 different AML groups based on the transcriptional profile.

Clinical data was collected in a standardized form, from a total of 26 Spanish centers that participated in the PETHEMA-FLUGAZA trial. Cytogenetic analysis was locally performed. Regarding molecular landscape, *NPM1*, *FLT3-ITD* and *CEPBA* mutation assessment was locally performed when possible. However, wide mutational data was retrospectively analyzed in a central laboratory with a myeloid NGS platform (Hospital Universitario 12 de Octubre, Madrid) (23) (**Figure 1A** **).** This clinical trial was conducted in accordance with the Declaration of Helsinki. Written informed consent was provided by all patients.

### Sample preparation and RNA sequencing

Bone marrow (BM) samples at diagnosis were characterized by multidimensional flow cytometry (MFC) in a central laboratory (CIMA, Centre for Applied Medical Research), and leukemic cells were purified by FACS. Co-Isolation protocol was performed to obtain DNA and RNA. Poly-A RNA was captured for further RNAseq protocol, while DNA was obtained from poly-A capture supernatant using SPRIselect beads (**Figure 1A** **).**

RNAseq was performed following MARS-seq protocol adapted for bulk RNAseq (24, 25) with minor modifications. Poly-A RNA was reverse-transcribed using poly-dT oligos carrying a 7 nt-index. Pooled samples were subjected to linear amplification by IVT. Resulting aRNA was fragmented and dephosphorylated. Ligation of partial Illumina adaptor sequences (24) was followed by a second reverse-transcription reaction. Full Illumina adaptor sequences were added during final library amplification. RNAseq libraries quantification was done with Qubit 3.0 Fluorometer (Life Technologies), and size profiles were examined using Agilent’s 4200 TapeStation System. Libraries were sequenced in an Illumina NextSeq 500 at a sequence depth of 10 million reads per sample. Raw reads were demultiplexed according to manufacturer’s instructions using bcl2fastq2 (v.2.20.0). Sequencing reads were aligned using STAR alignment tool (26) against hg38 reference genome. Counts were obtained using featureCounts from Rsubread R package, using ENSEMBL gene annotation (version 92). We used R (version 4.0.0) to perform hierarchical clustering and survival analysis. Results were visualized using R (**Figure 1A**).

### Statistical analysis

Overall survival (OS) was defined as the time since enrollment until death from any cause. Univariate and multivariate Cox regression analyses were calculated using R (version 4.0.0; The R Foundation, Vienna, Austria). Continuous variables are presented as means and standard deviations or as medians with ranges. Categorical variables are represented by frequencies and percentages. For all analyses, the P values were 2 tailed, and P < 0.05 was considered statistically significant. For the multivariate analysis, median values of Counts per Million (CPMs) were calculated for each gene, and then factorized as Low (*Gene* Low) or High (*Gene* High) according to the median value.

## Results

Baseline characteristics of patients included in the study are summarized in **Supplementary Table 1**. RNA sequencing (RNAseq) was performed on FACS sorted purified BM blasts obtained at diagnosis in 224 AML patients out of the 283 patients enrolled in the PETHEMA-FLUGAZA clinical trial (21) (112 of each arm) (**Figure 1A** **).** A total of 59 patients were excluded because of sample unavailability at diagnosis, assay failure, and/or bad sample quality. The median age at diagnosis was 75 years old. Median OS of the 224 patients was 5 months. Detailed treatment design, mutational landscape, ELN distribution and overall survival of patients are shown in **Figures S1A–D**.

Unsupervised hierarchical clustering identified 3 different groups based on the transcriptional profile (**Figure 1B**). There was no association between these transcriptional profiles and mutations in AML related genes, cytogenetics or ELN genetic risk categories such as the presence of *NPM1*, *FLT3-ITD*, *TP53*, *RUNX1* or *ASXL1* mutations. Survival analysis of the 3 transcriptomic groups did not show any differences, even though a trend to a better OS was identified for group 2 (**Figure 1C** **).**

Despite the dismal OS of this cohort, a group of elderly AML patients surviving beyond 12 months was identified (n=76). These long-term survivors were not characterized by a distinctive mutational or cytogenetic profile, and therefore we examined if there was a specific transcriptional signature associated with this group of patients. A differential expression analysis between patients surviving more or less than 12 months did not show any specific transcriptional signature either in the whole group or according to the treatment arm (**Figure S2**).

**Figure 2:**
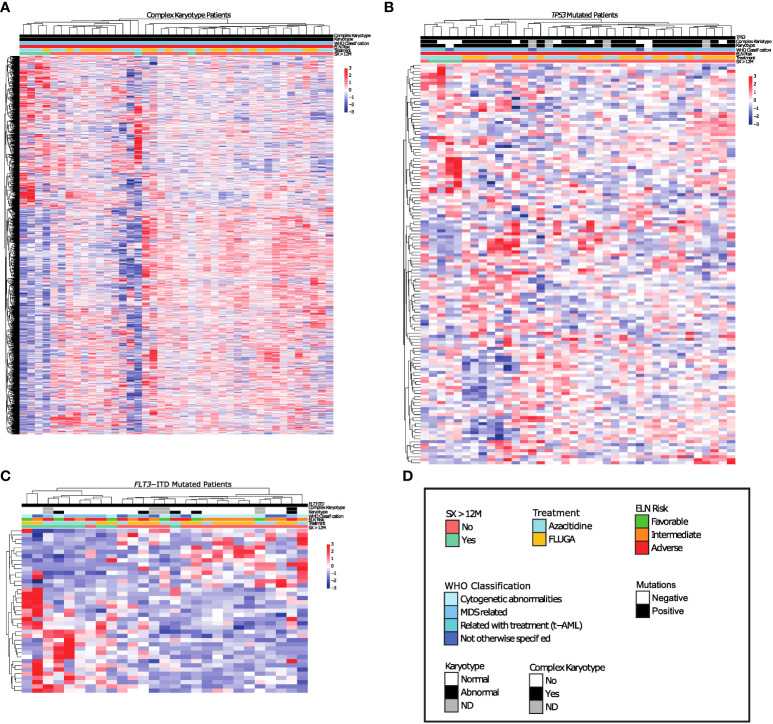
Long survival AML patients with complex Karyotype or *TP53* mutations show differential transcriptomic profile respect to non-long survival AML patients with these same genetic alterations. **(A-C)** Differential expression analysis between long survival AML patients and non-long survival AML patients with **(A)** Complex Karyotype, **(B)** *TP53* mutations. **(C)** FLT3-ITD mutations. **(D)** Legend.

**Figure 3:**
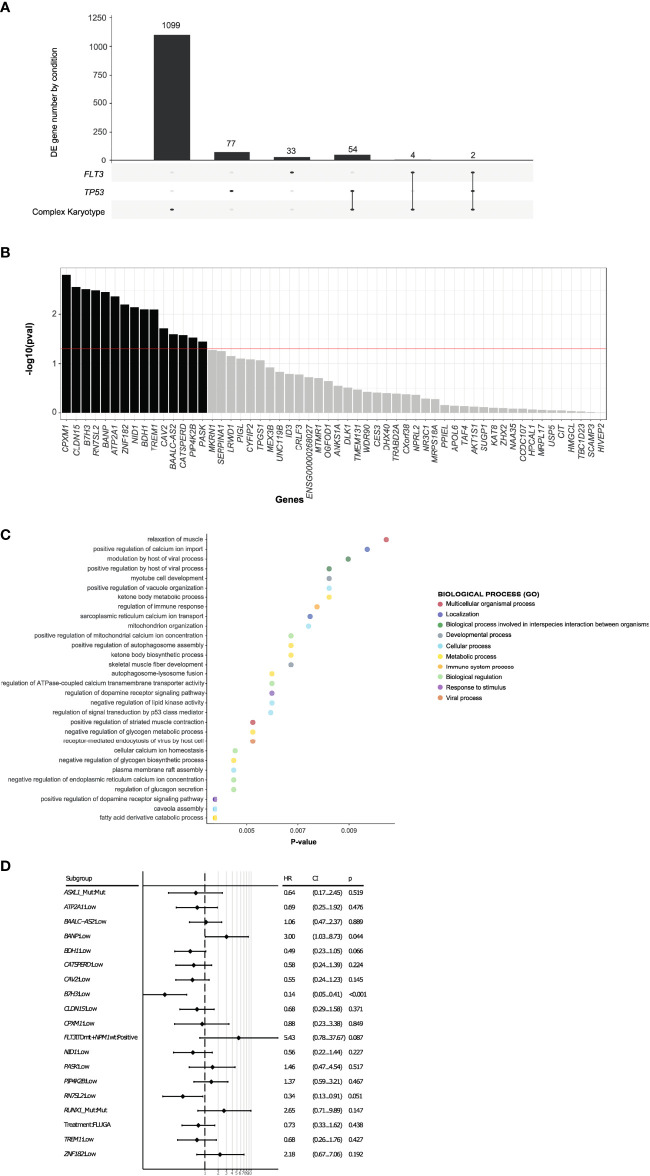
Expression of *B7H3 and BANP* genes are associated with overall survival of AML patients. **(A)** Differentially expressed genes between long-term survivors in the complex karyotype, *TP53* mutated and FLT*3-ITD* groups. **(B)** 15 differentially expressed genes shared by *TP53* mutated and complex karyotype long survival AML patients with impact on the univariate survival analysis. **(C)** GO study of the 15 differentially expressed genes in long survival AML patients with TP53 mutated or complex karyotype, with impact on the univariate survival analysis. **(D)** Multivariate analysis including differentially expressed 15 genes and ELN adverse category mutations such as *RUNX1*, *ASXL1, FLT3-ITD* with *NPM1 wt*.

We next analyzed the transcriptomic profiles for these long-term survivors according to the different genetic groups such as *FLT3-ITD*, *NPM1*, *TP53*, *RUNX1*, *TET2*, *IDH1/2* mutations and complex karyotype. Patients with mutations in *NPM1*, *RUNX1, IDH1/2* or *TET2* did not show a transcriptional profile associated with long-term survivors (**Figure S3**). However, a specific transcriptional profile was identified in long-term survivors with complex karyotype, *TP53* or *FLT3-ITD* mutations (**Figure 2**). When we focused at the differentially expressed genes between long-term survivors in the *FLT3-ITD*, *TP53* mutated and complex karyotype groups, we found that *TP53* mutated and complex karyotype groups showed most of the differentially expressed genes, 77 and 1099 respectively (**Figure 3A**). In this context, we focused our analysis in *TP53* mutated and complex karyotype patients. We found 56 differentially expressed genes shared in both groups of patients, out of which 15 genes (*CPXM1, CLDN15, B7H3, RN7SL2, BANP, ATP2A1, ZNF182, NID1, BDH1, TREM1, CAV2, BAALC-AS2, CATSPERD, PIP4K2B*, and *PASK*) were significantly associated with overall survival of AML patients on the univariate analysis *(* **Figure 3B**). Enrichment analysis was performed with those 15 genes in order to find altered pathways (**Figure 3C** and **Supplementary Table 2**).

**Figure 4:**
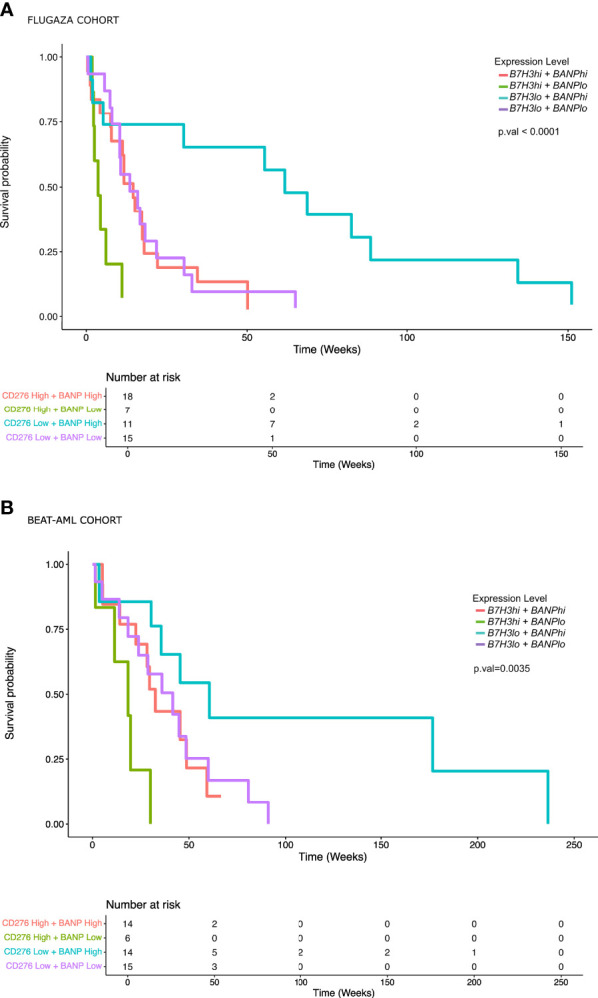
AML patients with low expression of *B7H3* and high expression of *BANP* show a significantly better overall survival. **(A)** Overall survival analysis in AML patients with *TP53* mutated or complex karyotype included in the PETHEMA-FLUGAZA trial using the expression of *B7H3* and *BANP* genes. **(B)** Overall survival analysis in AML patients included in BeatAML cohort using the expression of *B7H3* and *BANP* genes.

On the multivariate analysis including other adverse category mutations such as *RUNX1*, *ASXL1*, and *FLT3-ITDmt* with *NPM1wt*, as well as treatment arm, only the expression of *B7H3* and *BANP* was significantly associated with OS (**Figure 3D**). In fact, the expression of these 2 genes stratified patients with mutated *TP53* or complex karyotype into 3 groups with a different survival: patients with low expression of *B7H3* (CPM expression < 1.56 CPM) and high expression of *BANP* (CPM expression > 4.14 CPM) (*B7H3lo*/*BANPhi*) translated into a significantly better survival, whereas the opposite signature displayed a very short overall survival (*B7H3hi*/*BANPlo*) (MOS 1 month vs 14 months, p < 0.001). Patients with concordant expression profile (*B7H3lo*/*BANPlo*), (*B7H3hi*/*BANPhi*), presented with an intermediate prognosis (median OS 3.6 and 3.4 months respectively) (**Figure 4A**). Baseline characteristics of the three prognosis groups are summarized in **Supplementary Table 3**. Even though treatment arm did not have an impact in the multivariate analysis, (**Figure 3D**) we decided to confirm the prognostic value of our signature by taking each treatment arm separately, (**Figure S4**), confirming the same prognostic stratification. Finally, the prognostic value of the expression of *B7H3* and *BANP* was validated using the BeatAML independent cohort of AML patients (27) (**Figure 4B**), which includes also elderly patients intensively treated. Thus, low expression of *B7H3* (CPM expression, < 1.67 CPM) and high expression of *BANP* (CPM expression > 3.96 CPM) seem to identify a subset of patients with better outcome in the classical high-risk group of *TP53* mutated or complex karyotype elderly patients, including old AML patients treated with intensive chemotherapy.

## Discussion

AML in the elderly remains a clinical challenge. Currently in the clinical setting, the WHO and ELN risk stratification guidelines combine cytogenetic abnormalities and genetic mutations to establish optimal therapies for patients with AML. The development of novel RNA sequencing based prognostic scores for AML (28), including the integration of mutational and gene-expression data, have been found to add prognostic value to the current European Leukemia Net (ELN) risk classification as well as to identify new genomic subtypes. However, there is a need to identify patients that despite their general poor prognosis may experience a longer survival and/or can benefit from specific therapy.

In this study, exploiting RNAseq data obtained from a group of 224 AML patients homogeneously treated, we aimed to identify transcriptional biomarkers to identify patients with a different prognosis within ELN genetic risk groups. Although the unsupervised hierarchical clustering of the whole cohort identified 3 different transcriptional profiles, there were no association with classical AML related mutational and cytogenetic data. These findings might be consistent with the already well stablished heterogeneous nature of AML (10, 29). The lack of significant differences in survival between the 3 subgroups might be related with the small size of some of the transcriptional subgroups.

However, our results identified a unique transcriptomic signature in the typically adverse group with *TP53* mutation or complex karyotype, based on the expression of *B7H3* and *BANP* genes. This high risk group of patients have commonly a short survival of less than 12 months (30), and significantly worse if they are old or unfit (31). Conversely, we demonstrate that high risk AML patients with low expression of *B7H3* together with high expression of *BANP* gene display a significantly better overall survival than the whole group. This signature might modify the negative prognostic impact of TP53 or complex karyotype in AML patients.

Beside their role as biomarkers, both genes identified have been implicated in the pathogenesis of AML. *B7H3*, a transmembrane protein type I located in chromosome 15, is an immune checkpoint from the B7 family. Previous studies have identified high expression of *B7H3* as an adverse factor in multiple tumors, including AML (32, 33), having an *immunological function*, acting essentially as a coinhibitory immune checkpoint with an important role in immune editing and immune evasion. Prior studies have shown that *B7H3* generates an immunosuppressive tumor microenvironment, thus favoring immune surveillance evasion and promoting tumor progression (34). However, in recent years*, non-immunological functions* of *B7H3* seem to be even more important than the immunological ones for tumor aggressiveness. *B7H3* regulates migration, invasion and adhesion (34, 35), as well as promoting apoptosis resistance and chemoresistance in models of colorectal and breast cancer (33). Specifically in AML three different studies have addressed the implications of expression of *B7H3* (32, 36, 37). In the most comprehensive and integrative study including 625 patients with AML, they found that *B7H3* expression was essentially regulated by DNA methylation, and it was associated with old age, *TP53* mutations, and a poor outcome in four independent datasets. In line with these findings, another study conducted by Zhang, W (38) showed that *B7H3* knockdown in an AML cell line significantly decreased cell growth and enhanced chemosensitivity. We found a favorable outcome for high-risk patients with low *B7H3* expression, regardless of treatment arm. This is consistent with previously described functional implications of *B7H3*. AML patients with low *B7H3* expression could reflect a group with more active antitumor immunity, less aggressive AML cell properties and a more favorable chemosensitivity profile. Taken together, all these findings might explain why these initially high-risk patients do generally better in our cohort and enhance the potential of *B7H3* as a prognostic biomarker and possibly as a therapeutic target in high-risk AML.

*BANP* (BTG3 associated nuclear protein) is a nuclear matrix attachment region binding protein (MARBP) essential for nuclear matrix binding that has been implicated in cancer. MARBPs facilitate a correct chromatin assembly necessary for the normal gene replication and transcription (39). Thus, perturbations in these proteins might lead to an incorrect chromatin folding and aberrant replication and transcriptional programs, promoting genomic instability and oncogenesis (40, 41). Kaul et al. conducted the first study in mouse melanoma cells (42), showing that ectopic expression of *SMAR1* (murine homolog of *BANP*) promoted cell arrest. Subsequent studies have been carried out mainly in breast cancer models (43, 44). *BANP* exerts its antitumor activity through the modulation of crucial transcription factors such as p53 and NFκβ (45). These interactions take place through the formation of complexes with histone deacetylase (HDAC1). In addition, *BANP* regulates the TGFβ pathway by inducing the expression of SMAD7, an inhibitory SMAD that negatively regulates the TGFβ pathway. These studies reflect the important role of *BANP* as a tumor suppressor gene in cancer and may be consistent with our findings in which higher levels of *BANP* expression were associated with a favorable outcome in high-risk AML patients. In that sense, therapeutic approaches addressing the stabilization of *BANP* expression may be warranted.

In conclusion, we performed RNAseq in 224 elderly AML patients homogeneously treated, with the aim to define new prognostic groups. We identified the expression of *B7H3* and *BANP* genes as unique transcriptomic biomarkers, revealing a long survival group within *TP53* mutated or complex karyotype AML patients. As a potential limitation of our study, we acknowledge that *TP53* mutation assessment was performed with NGS, therefore information regarding *multihit* TP53 mutation was not available. According to our findings, *B7H3lo/BANPhi* patients have a clinical course more similar to a low-risk genetic group, and this signature might reduce the negative prognostic impact of TP53 or complex karyotype in AML patients. These two genes might serve as prognostic biomarkers and functional studies should address its utility as therapeutic targets in AML.

## Data availability statement

The data presented in the study are deposited in Gene Expression Omnibus repository, with accession number:GSE208218.

## Ethics statement

The studies involving human participants were reviewed and approved by PETHEMA. The patients/participants provided their written informed consent to participate in this study.

## Author contributions

SaV and BA wrote the manuscript. FP, XA, BP and AA-P revised and modified the manuscript. BA and SaV performed statistical analysis. DM-C, JB, SuV, LA, MT, PiM, JS, CS, PH, MC, AA-P, BP, JM-L and PaM provided study material and/or patients. AU and SaV prepared RNA-Seq libraries. All authors contributed to the article and approved the submitted version.

## Funding

The authors acknowledge all investigators involved in the PETHEMA-FLUGAZA phase 3 clinical trial. This work was supported by the CIBERONC (CB16/12/00369, CB16/12/00233, CB16/12/00489, and CB16/12/00284), Instituto de Salud Carlos III/Subdirección General de Investigación Sanitaria, Fondo de Investigación en Salud (FIS no. PI16/01661 and PI16/00517), Government of Navarra (Project AGATA, 0011-1411-2020-000013).

## Conflict of interest

RA: Membership on an entity´s Board of Directors advisory committees: Incyte Corporation, Astellas; Honoraria: Novartis, Celgene and Incyte. MT: declares honoraria for lectures from Celgene, Pfizer, Novartis, Janssen, Merck Sharp & Dohme (MSD), Daiichi, and Servier SL, and membership on advisory boards with Celgene, Novartis, Roche, and Astellas. JS: declares honoraria for lectures, and membership on advisory boards with, Daiichi Sankyo, Pfizer, Celgene, Novartis, Roche, and Amgen. BP: served as a consultant for and received honoraria from Adaptive, Amgen, Becton Dickinson, Bristol Myers Squibb/Celgene, GSK, Janssen, Roche, Sanofi, and Takeda; and received research support from Bristol Myers Squibb/Celgene, GSK, Roche, Sanofi, and Takeda JM-L: declares honoraria for lectures from, and membership on advisory boards with, Janssen, BMS, Sanofi, Novartis, Incyte, Roche, and Amgen; and membership on the boards of directors of Hosea and Altum Sequencing. JFS-M: reports Consultancy, membership on an entity´s Board of Directors advisory committees: AbbVie, Amgen, Bristol-Myers Squibb, Celgene, GlaxoSmithKline, Janssen, Karyopharm, Merck Sharpe & Dohme, Novartis, Regeneron, Roche, Sanofi, SecuraBio, Takeda. FP: Honoraria and research funding: Oryzon, Janssen, BMS-Celgene. PM: declares Consultancy, membership on an entity´s Board of Directors advisory committees, research funding, speaker’s bureau: Celgene, Sanofi, Incyte, Karyopharm, Novartis, Stemline/Menarini, Agios, Astellas Pharma, Daiichi Sankyo; Membership on an entity´s Board of Directors advisory committees: Pfizer, Teva, AbbVie; Research Funding, Speakers Bureau: Janssen; Consultancy: Tolero Pharmaceutical, Forma Therapeutics, Glycomimetics.

The remaining authors declare no competing financial interests.

## Publisher’s note

All claims expressed in this article are solely those of the authors and do not necessarily represent those of their affiliated organizations, or those of the publisher, the editors and the reviewers. Any product that may be evaluated in this article, or claim that may be made by its manufacturer, is not guaranteed or endorsed by the publisher.

## Supplementary material

Supplementary Figure 1 Detailed treatment design and mutational landscape of patients. **(A)** Scheme showing the two arms of treatment for AML patients in the clinical trial, FLUGA treatment arm (top) or AZA treatment arm (bottom). **(B)** Mutational landscape of AML patients included in the FLUGAZA clinical trial.

Supplementary Figure 2 AML patients surviving more than 12 months do not display any specific transcriptional signature. **(A-C)** Differential expression analysis between AML patients surviving more or less than 12 months **(A)** in the whole group, **(B)** in the AZA treatment arm or **(C)** in the FLUGA treatment arm. SX: more than 12 months of survival.

Supplementary Figure 3 Long survival AML patients with mutations in *NPM1*, *IDH1/2*, *TET2* or *RUNX1* do not show any differential transcriptomic profile respect to non-long survival AML patients with these same genetic alterations. **(A-D)** Differential expression analysis between long survival AML patients and non-long survival AML patients with **(A)** *NPM1* mutations, **(B)** *RUNX1* mutations, **(C)** *IDH1/2* mutations or **(D)** *TET*2 mutations.

Supplementary Figure 4 Overall survival analysis in AML patients with *TP53* mutated or complex karyotype included in the PETHEMA-FLUGAZA trial using the expression of *B7H3* and *BANP* genes according to their treatment arm. **(A)** AZA arm. **(B)** FLUGA arm.

Supplementary Table 1 Baseline characteristics of patients included in the FLUGAZA-PETHEMA clinical trial in whom RNASeq was performed.

Supplementary Table 2 Gene ontology pathways and biological processes associated with the 15 differentially expressed genes with survival impact on the univariate analysis in mutated *TP53* or complex karyotype groups.

Supplementary Table 3 Baseline characteristics of 51 patients of the cohort with the selected genetic signature according to the obtained prognosis group.
