# Supplementary figures and images for: The transcriptomic landscape of elderly acute myeloid leukemia identifies B7H3 and BANP as a favorable signature in high-risk patients
Source: Front Oncol. 2022 Nov 24;12:1054458. doi: 10.3389/fonc.2022.1054458 (PMC9729799; doi:10.3389/fonc.2022.1054458)

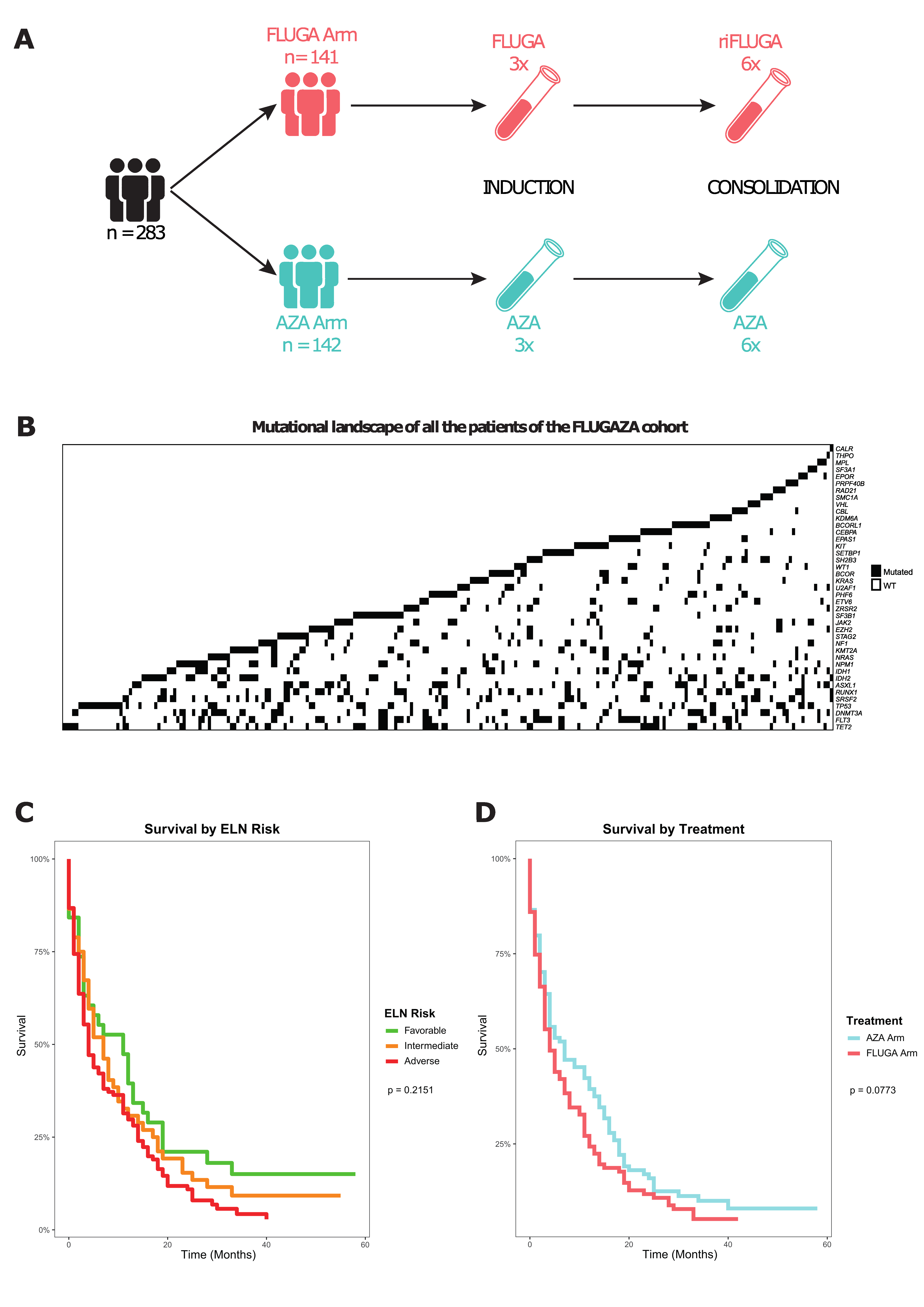

Supplement: Supplementary Figure 1 — Detailed treatment design and mutational landscape of patients. (A) Scheme showing the two arms of treatment for AML patients in the clinical trial, FLUGA treatment arm (top) or AZA treatment arm (bottom). (B) Mutational landscape of AML patients included in the FLUGAZA clinical trial. [file Image_1.tiff]

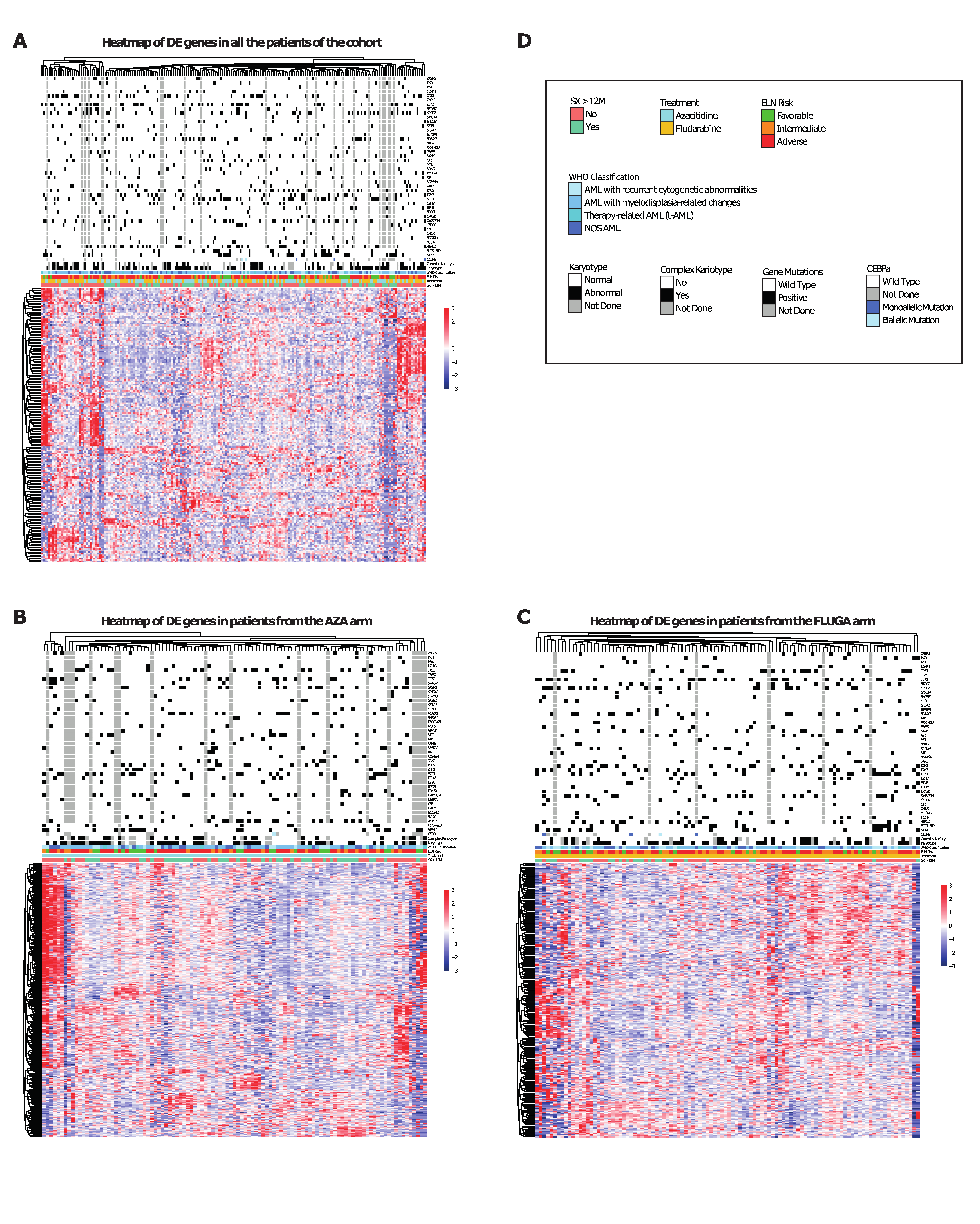

Supplement: Supplementary Figure 2 — AML patients surviving more than 12 months do not display any specific transcriptional signature. (A-C) Differential expression analysis between AML patients surviving more or less than 12 months (A) in the whole group, (B) in the AZA treatment arm or (C) in the FLUGA treatment arm. SX: more than 12 months of survival. [file Image_2.tiff]

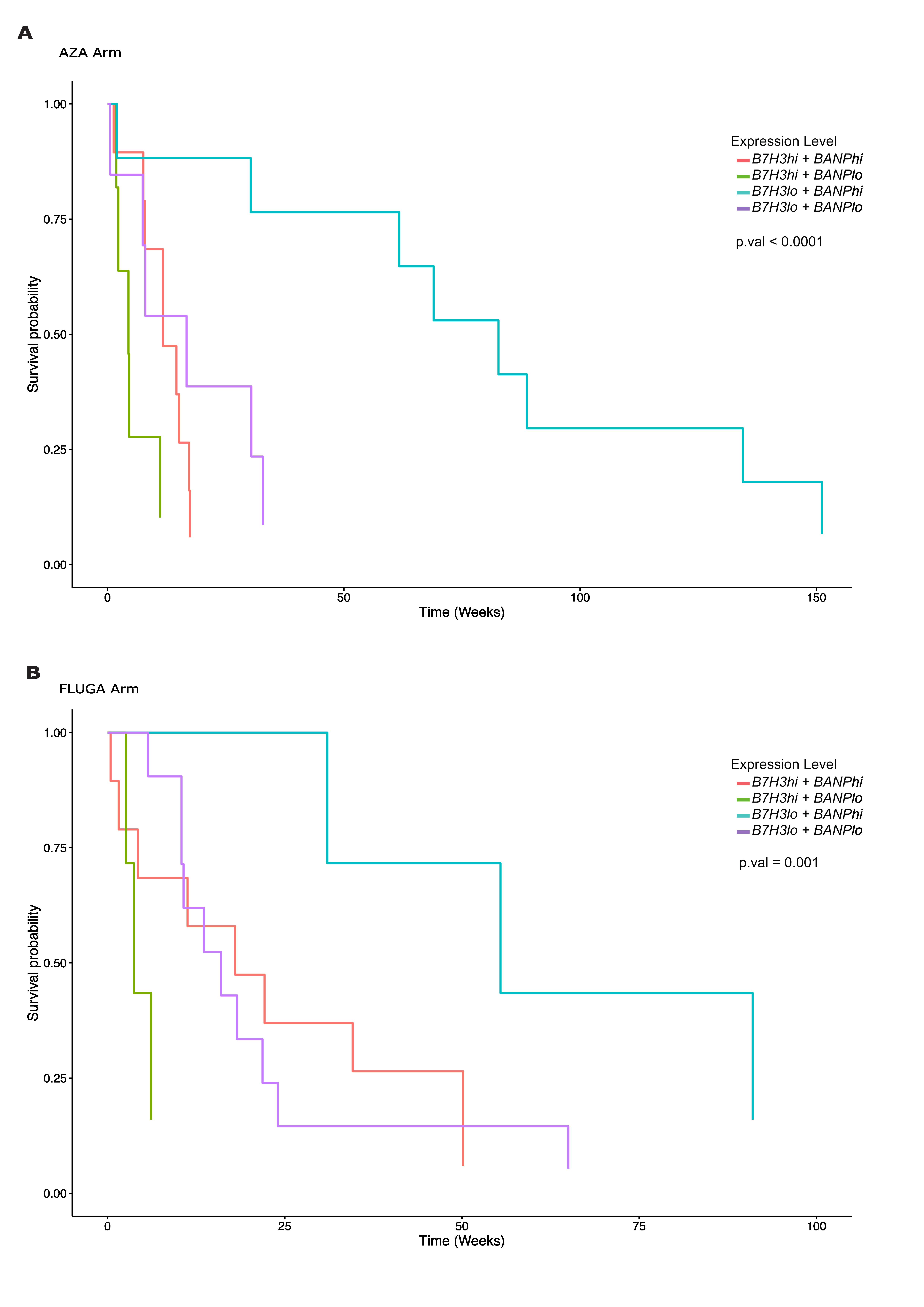

Supplement: Supplementary Figure 4 — Overall survival analysis in AML patients with TP53 mutated or complex karyotype included in the PETHEMA-FLUGAZA trial using the expression of B7H3 and BANP genes according to their treatment arm. (A) AZA arm. (B) FLUGA arm. [file Image_4.tiff]
