# Supplementary material for: The transcriptomic landscape of elderly acute myeloid leukemia identifies B7H3 and BANP as a favorable signature in high-risk patients
Source: Front Oncol. 2022 Nov 24;12:1054458. doi: 10.3389/fonc.2022.1054458 (PMC9729799; doi:10.3389/fonc.2022.1054458)
Supplement: Supplementary Table 1 — Baseline characteristics of patients included in the FLUGAZA-PETHEMA clinical trial in whom RNASeq was performed. [file Table_1.pdf]

TABLE 1. Baseline Characteristics of patients in the RNA-Seq cohort of PETHEMA-FLUGAZA

**Characteristic**	**Whole series (n=224)**	**FLUGA (n=112)**	**AZA (n= 112)**	**p**
Age at diagnosis (years)	75 (65-90)	76 (65-88)	74 (65-90)	0.226
Sex (male), n (%)	127 (56.7%)	58 (51.7%)	69 (61.6%)	0.178
WBC ≥ 100 x 10^9/L, n (%)	9 (4%)	5 (4.5%)	4 (3.6%)	1
BM blasts (%)	52 (33-76)	51 (30-78)	52.5 (34-73)	0.929
Cytogenetics				0.777
Normal karyotype, n (%)	91 (40.6%)	47 (42%)	44 (39.3%)	
Abnormal karyotype, n (%)	58 (25.9%)	31 (27.7%)	27 (24.1%)	
Complex karyotype, n (%)	42 (18.8%)	19 (17%)	23 (20.5%)	
Missing, n (%)	33 (14.7%)	15 (13.3%)	18 (16.1%)	
AML subtype				0.737
De novo, n (%)	94 (42%)	47 (42%)	47 (42%)	
AML-MRC, n (%)	113 (50.4%)	55 (49.1%)	58 (51.8%)	
t- AML, n (%)	17 (7.6%)	10 (8.9%)	7 (6.2%)	
ELN 2017 risk				0.275
Favourable, n (%)	40 (17.9%)	18 (16.1%)	22 (19.6%)	
Intermediate, n (%)	54 (24.1%)	33 (29.4%)	21 (18.8%)	
Adverse, n (%)	123 (54.9%)	57 (50.9%)	66 (58.9%)	
Missing, n (%)	7 (3.1%)	4 (3.6%)	3 (2.7%)	

Abbreviations: AML, acute myeloid leukemia; AML-MRC acute myeloid leukemia with myelodysplasia-related changes; AML-t, acute myeloid leukemia related to therapy; AZA, azacytidine; FLUGA, fludarabine, cytarabine and filgrastim schedule; BM, bone marrow; ELN, European Leukemia Net; LDH, lactic dehydrogenase; WBC, white blood cell count.
Values for continuous variables are expressed as median (range) except otherwise indicated.
