# Supplementary material for: The transcriptomic landscape of elderly acute myeloid leukemia identifies B7H3 and BANP as a favorable signature in high-risk patients
Source: Front Oncol. 2022 Nov 24;12:1054458. doi: 10.3389/fonc.2022.1054458 (PMC9729799; doi:10.3389/fonc.2022.1054458)
Supplement: Supplementary Table 2 — Gene ontology pathways and biological processes associated with the 15 differentially expressed genes with survival impact on the univariate analysis in mutated TP53 or complex karyotype groups. [file Table_2.pdf]

Term	Genes	Biologic process	P-value	Adjusted P-value
fatty acid derivative catabolic process (GO:1901569)	BDH1	Metabolic process	0.00374469708393798	0.04345432293940626
caveola assembly (GO:0070836)	CAV2	Cellular process	0.00374469708393798	0.04345432293940626
positive regulation of dopamine receptor signaling pathway (GO:0060161)	CAV2	Response to stimulus	0.00374469708393798	0.04345432293940626
regulation of glucagon secretion (GO:0070092)	PASK	Biological Regulation	0.004492068393214533	0.04345432293940626
negative regulation of endoplasmic reticulum calcium ion concentration (GO:0032471)	ATP2A1	Biological Regulation	0.004492068393214533	0.04345432293940626
plasma membrane raft assembly (GO:0044854)	CAV2	Cellular process	0.004492068393214533	0.04345432293940626
negative regulation of glycogen biosynthetic process (GO:0045719)	PASK	Metabolic process	0.004492068393214533	0.04345432293940626
cellular calcium ion homeostasis (GO:0006874)	CAV2;ATP2A1	Biological Regulation	0.004547996709660412	0.04345432293940626
receptor-mediated endocytosis of virus by host cell (GO:0019065)	CAV2	Viral process	0.005238916894144343	0.04345432293940626
negative regulation of glycogen metabolic process (GO:0070874)	PASK	Metabolic process	0.005238916894144343	0.04345432293940626
positive regulation of striated muscle contraction (GO:0045989)	ATP2A1	Multicellular organismal process	0.005238916894144343	0.04345432293940626
regulation of signal transduction by p53 class mediator (GO:1901796)	PIP4K2B;BANP	Cellular process	0.0059381164344082405	0.04345432293940626
negative regulation of lipid kinase activity (GO:0090219)	PIP4K2B	Cellular process	0.0059852428364459065	0.04345432293940626
regulation of dopamine receptor signaling pathway (GO:0060159)	CAV2	Response to stimulus	0.0059852428364459065	0.04345432293940626
regulation of ATPase-coupled calcium transmembrane transporter activity (GO:1901894)	ATP2A1	Biological Regulation	0.0059852428364459065	0.04345432293940626
autophagosome-lysosome fusion (GO:0061909)	PIP4K2B	Metabolic process	0.0059852428364459065	0.04345432293940626
skeletal muscle fiber development (GO:0048741)	CAV2	Developmental process	0.006731046493869444	0.04345432293940626
ketone body biosynthetic process (GO:0046951)	BDH1	Metabolic process	0.006731046493869444	0.04345432293940626
positive regulation of autophagosome assembly (GO:2000786)	PIP4K2B	Metabolic process	0.006731046493869444	0.04345432293940626
positive regulation of mitochondrial calcium ion concentration (GO:0051561)	ATP2A1	Biological Regulation	0.006731046493869444	0.04345432293940626
mitochondrion organization (GO:0007005)	CAV2;ATP2A1	Cellular process	0.0074167494874084125	0.04345432293940626
sarcoplasmic reticulum calcium ion transport (GO:0070296)	ATP2A1	Localization	0.0074763281564485876	0.04345432293940626
regulation of immune response (GO:0050776)	TREM1;CD276	Immune system process	0.0077472708863843154	0.04345432293940626
ketone body metabolic process (GO:1902224)	BDH1	Metabolic process	0.008221088123671455	0.04345432293940626
positive regulation of vacuole organization (GO:0044090)	PIP4K2B	Cellular process	0.008221088123671455	0.04345432293940626
myotube cell development (GO:0014904)	CAV2	Developmental process	0.008221088123671455	0.04345432293940626
positive regulation by host of viral process (GO:0044794)	CAV2	Interspecies interaction between organisms	0.008221088123671455	0.04345432293940626
modulation by host of viral process (GO:0044788)	CAV2	Interspecies interaction between organisms	0.008965326705510297	0.04575408111777669
positive regulation of calcium ion import (GO:0090280)	ATP2A1	Localization	0.009709044215672634	0.047897951463985
relaxation of muscle (GO:0090075)	ATP2A1	Multicellular organismal process	0.0104522409709381	0.049901021409639965
