# Supplementary material for: The transcriptomic landscape of elderly acute myeloid leukemia identifies B7H3 and BANP as a favorable signature in high-risk patients
Source: Front Oncol. 2022 Nov 24;12:1054458. doi: 10.3389/fonc.2022.1054458 (PMC9729799; doi:10.3389/fonc.2022.1054458)
Supplement: Supplementary Table 3 — Baseline characteristics of 51 patients of the cohort with the selected genetic signature according to the obtained prognosis group. [file Table_3.docx]

| **Characteristics** | **Good Prognosis Group (n=11)** | **Intermediate Prognosis Group (n=33)** | **Bad Prognosis Group (n=7)** | **p** |
| --- | --- | --- | --- | --- |
| Age at diagnosis | 74 [68-84] | 77 [68-88] | 78 [69-90] | 0.291 |
| Sex (male), n (%) | 4 (36.4%) | 19 (57.6%) | 2 (28.6%) | 0.241 |
| Cytogenetics  Normal Karyotype, n (%)  Abnormal Karyotype, n (%)  Missing, n (%) | 1 (9.1%)  9 (81.8%)  1 (9.1%) | 2 (6.1%)  27 (81.8%)  4 (12.1%) | 0 (0%)  7 (100%)  0 (0%) | 0.353 |
| Complex Karyotype  Yes, n (%)  No, n(%)  Missing, n (%) | 7 (63.6%)  3 (27.3%)  1 (9.1%) | 23 (69.7%)  6 (18.4%)  4 (12.1%) | 6 (85.7%)  1 (14.3%)  0 (0%) | 0.801 |
| TP53 (Yes), n (%) | 7 (63.6%) | 27 (81.8%) | 7 (100%) | 0.157 |
| AML Subtype  De novo, n (%)  AML-MRC, n (%)  t-AML, n (%) | 1 (9.1%)  8 (72.7%)  2 (18.2%) | 3 (9.1%)  28 (84.8%)  2 (6.1%) | 1 (14.3%)  5 (71.4%)  1 (14.3%) | 0.845 |

Abbreviations: AML, acute myeloid leukemia; AML-MRC acute myeloid leukemia with myelodysplasia-related changes; AML-t, acute myeloid leukemia related to therapy.

Values for continuous variables are expressed as median (range) except otherwise indicated.
